# Novel links of ATG4 proteases to cytoskeletal adapters of the obscurin protein family

**DOI:** 10.1080/15548627.2025.2572530

**Published:** 2025-10-15

**Authors:** Virginia Actis Dato, Kyohei Fujita, Stephan Lange

**Affiliations:** a Division of Cardiovascular Medicine, UC San Diego, La Jolla, CA, USA; b Institute of Advanced Biomedical Engineering and Science, Tokyo Women’s Medical University, Tokyo, Japan; c Department of Biomedicine, Aarhus University, Aarhus, Denmark; d STENO Diabetes Center Aarhus, Aarhus University Hospital, Aarhus, Denmark

**Keywords:** ATG4, ATG8, mitophagy, OBSCN, OBSL1

## Abstract

The obscurin family, containing the giant protein OBSCN (obscurin, cytoskeletal calmodulin and titin-interacting RhoGEF) and its closely related OBSL1 (obscurin like cytoskeletal adaptor 1) as well as SPEG (striated muscle enriched protein kinase) are a group of intracellular proteins that contain serially linked immunoglobulin (Ig) and fibronectin type III (Fn3) domains, along with signaling modules such as protein kinase domains. Hence, obscurins harbor multi-faceted roles for the architecture and organization of cell- and organelle membrane proteins. Besides mediating cellular signaling and promoting protein homeostasis, obscurin proteins are also proposed to act as versatile cytoskeletal linkers. Due to their close homology, many functions for OBSCN are evolutionary conserved in OBSL1 and SPEG. However, their expression patterns differ widely, with OBSL1 being ubiquitously expressed in all cell types, while OBSCN and SPEG are more restricted to cross-striated muscles. Recent evidence indicates that autophagy-linked peptidases of the ATG4 family interact with the cytoskeletal adapter proteins OBSL1 and OBSCN. Peptidases of the ATG4 family process Atg8-family proteins (e.g. LC3) in their immature state (i.e. as pro-peptides like pro-LC3) or their bioactive lipidated state (i.e. LC3-II) and facilitate their conversion to the delipidated state (i.e. LC3-I). Loss of interaction between ATG4 peptidases and obscurin family proteins affects cellular macroautophagy/autophagy and mitophagy, leading in situations of cellular stress to depletion of ATG4 and accumulation of the lipidated state for Atg8-family proteins (e.g. LC3-II).

## Novel interactions of obscurin proteins with ATG4 proteases

**Figure 1.**
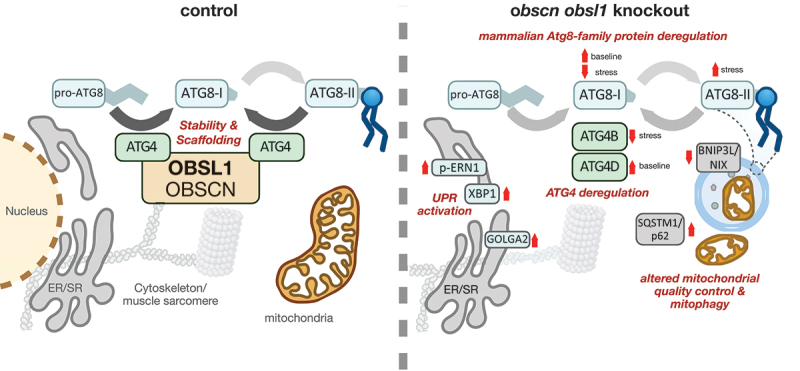
Obscurin proteins (OBSCN and OBSL1) regulate autophagy by scaffolding and stabilizing ATG4 cysteine peptidases, which are essential for processing of Atg8-family proteins. Deletion of *Obscn* and *Obsl1* disrupts these processes, resulting in the deregulation of ATG4 and Atg8-family proteins. This dysregulation leads to impaired autophagy and mitophagy, as also visible in accumulation of SQSTM1/p62 in double-knockout (dKO) hearts. Mitochondria in dKO hearts appear additionally reduced in size and show decreased expression of mitophagy-related proteins such as BNIP3L/NIX. Moreover, elevated phosphorylation of ERN1/IRE1α, along with increased levels of XBP1 and GOLGA2/GM130, point to activation of the unfolded protein response (UPR) in the absence of OBSCN and OBSL1. ATG8: mammalian Atg8-family protein.

Recently, we identified ATG4 as novel interaction partner for OBSL1 [1] (Figure 1). While originally only shown for the mitochondrially-linked ATG4D, the interaction with OBSL1 appears conserved for more members of the ATG4 peptidase family, including the more versatile ATG4B. Binding of the peptidase domain in ATG4 is mediated by the two C-terminal Ig-domains in OBSL1. Although not tested for the de-lipidase function of the enzymes, association of ATG4 with OBSL1 had no noticeable effect on the in-vitro processing of Atg8-family pro-peptides (e.g. pro-LC3, pro-GABARAPL1). However, loss of OBSL1 in non-muscle cells leads to accumulation of ATG4D but not ATG4B at baseline.

In cross-striated muscle cells, such as cardiomyocytes, some functions for OBSL1 are conserved in OBSCN, including the anchorage of the obscurin family to the muscle sarcomeres. Loss of OBSL1 alone in cardiomyocytes does not affect ATG4D, but additional deletion of *Obscn* significantly increases ATG4D levels. This finding suggests that OBSCN also contains a conserved binding site for ATG4-family proteins, indicating functional redundancy between OBSL1 and OBSCN.

In times of cellular stress, such as during CCCP treatment of cells that decouples the electron transport chain and induces mitophagy, ATG4B levels are markedly reduced in *obscn obsl1* double-knockout (dKO) cardiomyocytes, leading to the depletion in the pool of available LC3-I. Due to their roles in the formation and regulation of CUL (cullin)-associated ubiquitin E3-ligase complexes, our data suggest that obscurin proteins may provide scaffolding or stabilizing functions that modulate cellular ATG4 levels, thereby affecting Atg8-family protein availability, recycling and proteostasis, and indirectly influencing cellular autophagy.

## Deletion of *obscn* and *obsl1* affects autophagy/mitophagy and cellular stress-response mechanisms

What are general effects of obscurin protein family loss on the autophagy system? In steady state, hearts of *obscn obsl1* dKO mice exhibit signs reminiscent of deregulated autophagy, with elevated levels of SQSTM1/p62 (sequestosome 1) and autophagic double-membraned vesicles visible in electron micrographs. Particularly affected by the lack of OBSCN and OBSL1 are mitochondria and cardiomyocyte mitophagy. Cardiac mitochondria appear to be overall decreased in size, and proteins involved in mitophagy such as BNIP3L/NIX are significantly decreased in dKO hearts. Whereas expression of Oxphos proteins is unaltered at baseline, induction of mitophagy by CCCP results in increased degradation of electron transport chain proteins. Besides the reduction in LC3-I levels, ATG16L1 protein and phosphorylation levels are also severely decreased in dKO cardiomyocytes. These findings support a role for OBSCN and OBSL1 in maintaining mitochondrial capacity during cellular stress.

The impact of obscurin family proteins may extend to the endoplasmic reticulum (ER), as increased ERN1/IRE1α phosphorylation and elevated XBP1 and GOLGA2/GM130 levels are observed in dKO hearts. These changes point to activation of the unfolded protein response (UPR), possibly secondary to compromised autophagy/mitophagy and ER-mitochondrialcrosstalk. Thus, OBSCN and OBSL1 appear to maintain proteostasis through multiple mechanisms.

In conclusion, we propose a model in which obscurin proteins indirectly regulate autophagy and mitophagy, by modulating the stability of ATG4 cysteine peptidases that play essential roles for the processing of Atg8-family proteins. Our findings suggest that loss of OBSCN and OBSL1 compromises this process, impairing mitochondrial quality control and mitophagy as well as autophagic flux, and linking these cytoskeletal proteins to cellular stress resilience. Importantly, disruptions to autophagy and mitophagy are most pronounced in OBSCN OBSL1 loss-of-function models, indicating functional redundancy between both proteins, such as in cross-striated muscle cells.

How OBSCN or OBSL1 contribute to human disease remains to be further explored. However, given the essential role of mitophagy in preserving mitochondrial structure and function in diseases such as heart failure with preserved ejection fraction/HFpEF or various metabolic disorders, further investigation into obscurin family proteins and their links to maintaining proteostasis by modulating the ubiquitin-proteasome or the autophagy system is warranted.

## Data Availability

There are no datasets associated with this submission. Datasets associated with the original manuscript are accessible via Gene Expression Omnibus (accession number GSE283745) or ProteomeXchange (accession number PXD058697). Obscurin and Obsl1 gene targeted mice are available at The Jackson Laboratory (IMSR_JAX:035303 and IMSR_JAX:035302, respectively).
